# AHNAK Contributes to Hepatocellular Carcinoma Growth by Interacting with IGF-1R

**DOI:** 10.3390/molecules27248680

**Published:** 2022-12-08

**Authors:** Kang Li, Ke Song, Yuli Hou, Yuan Tian, Huijuan Wang, Libo Sun, Ang Li, Yonghong Zhang

**Affiliations:** 1 Biomedical Information Center, Beijing You’An Hospital, Capital Medical University, Beijing 100069, China; 2 Department of Histology and Embryology, School of Basic Medical Sciences, Capital Medical University, Beijing 100069, China; 3 Department of Clinical Laboratory, Xuanwu Hospital, National Clinical Research Center for Geriatric Diseases, Capital Medical University, Beijing 100053, China; 4 Beijing Institute of Hepatology, Beijing You’An Hospital, Capital Medical University, Beijing 100069, China; 5 Department of Hepatobiliary Surgery, Beijing You’An Hospital, Capital Medical University, Beijing 100069, China

**Keywords:** neuroblast differentiation-associated protein AHNAK, hepatocellular carcinoma (HCC), insulin-like growth factor 1 receptor (IGF-1R), interactome, field cancerization

## Abstract

Neuroblast differentiation-associated protein AHNAK, a large structural scaffold protein, remains mysterious in biological processes. AHNAK plays a suppressive or progressive role in different types of cancers. To investigate the role of the AHNAK in hepatocellular carcinoma (HCC), cell viability assays were performed to determine the cell proliferation of the stable AHNAK-knockdown HepG2 cell line; co-immunoprecipitation (Co-IP) and liquid chromatography coupled with tandem mass spectrometry (LC-MS/MS) were performed on HCC and matched paracancerous (MPC) tissues. The Metascape platform was used for enrichment analyses; the “ComplexHeatmap” package was applied for cluster analyses and visualization. Co-IP, Western botting and immunofluorescence double staining were performed to assess the interactions between AHNAK and insulin-like growth factor 1 receptor (IGF-1R). AHNAK silencing reduced the viability of HepG2 cells; the interactome in HCC and MPC tissues enriched 204 pathways and processes, which partially reflected the signature of HCC field cancerization. AHNAK could co-localize and interact with IGF-1R. These results suggested that the AHNAK complex contributes to HCC growth, potentially by interacting with IGF-1R.

## 1. Introduction

Hepatocellular carcinoma (HCC) is one of the most common and lethal tumors worldwide and the most common type of primary liver cancer, accounting for 75–85% of cases [1]. Despite several signaling pathways having been identified to be related with the progression of HCC, the molecular mechanism of HCC development remains complicated. Adjacent tumor tissues are not completely normal tissues [2], which is known as field cancerization [3,4]. Although histologically normal, they share some common molecular alterations with tumors. Field cancerization has been shown to affect circRNA expression profiles in gastric cancer [3]. This understanding might also provide new enlightenment for the study of the occurrence and development of HCC.

Neuroblast differentiation-associated protein AHNAK, also known as desmoyokin [5], is a large structural scaffold protein (molecular mass >620 kDa) that remains mysterious and plays diverse roles in the biological processes of various cancers [6]. As reported in the literature, AHNAK suppressed the proliferation and invasion of triple-negative breast cancer via different signaling pathways [7]. AHNAK also suppressed the progress of ovarian cancer by impairing the canonical Wnt/β-catenin pathway [8]. Similar results occurred in colorectal cancer [9] and melanoma [10]. However, a high level of AHNAK was associated with poor outcome in pancreatic ductal adenocarcinoma (PDAC) [11] and laryngeal carcinoma [12]. It could enhance the metastasis of lung cancer via epithelial–mesenchymal transition [13] and promote migration and invasion in mesothelioma [14]. In liver cancer, our previous research found that the mRNA expression of AHNAK was elevated in liver cancer tissues and the methylation level of AHNAK decreased from liver disease to HCC [15]. However, the molecular mechanism via which AHNAK is involved in HCC progression is unclear.

Therefore, this study utilized co-immunoprecipitation (Co-IP) and liquid chromatography coupled with tandem mass spectrometry (LC-MS/MS) to analyze the AHNAK interactome in liver cancer and matched paracancerous (MPC) tissues, aiming to investigate the AHNAK involving signaling pathways. Our findings indicate that AHNAK could serve as a potential candidate targeting the IGF-1R signaling pathway to promote the development of HCC.

## 2. Results

### 2.1. Specific Knockout of AHNAK Protein Can Inhibit the Proliferation of HepG2 Cells

**Figure 1:**
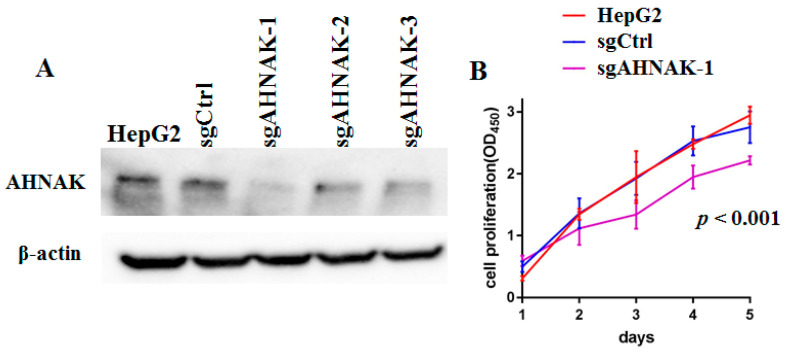
AHNAK expression was essential for HepG2 cell growth: (**A**) Results of Western blotting analysis of AHNAK expression in sgCtrl, sgAHNAK-1, sgAHNAK-2 and sgAHNAK-3 HepG2 cells, and HepG2 cells. (**B**) Cell counting kit-8 assays were used to determine cell viability in sgCtrl and sgAHNAK-1 HepG2 cells, and HepG2 cells (n = 3).

To determine the effect of AHNAK on HepG2 cell proliferation, modified lentiviral AHNAK vectors were used to knock out the expression of AHNAK in HepG2 cells. The expression of AHNAK was markedly diminished with the application of sgAHNAK-1, sgAHNAK-2 and sgAHNAK-3 lentiviruses (Figure 1A). Due to the higher efficiency, the sgAHNAK-1 lentivirus was utilized in our subsequent experiments. Cell counting kit-8 (CCK8) assays demonstrated that AHNAK silencing led to reduced viability of HepG2 cells (Figure 1B).

### 2.2. Expression Analysis of AHNAK Protein in Liver Cancer Tissues

**Figure 2:**
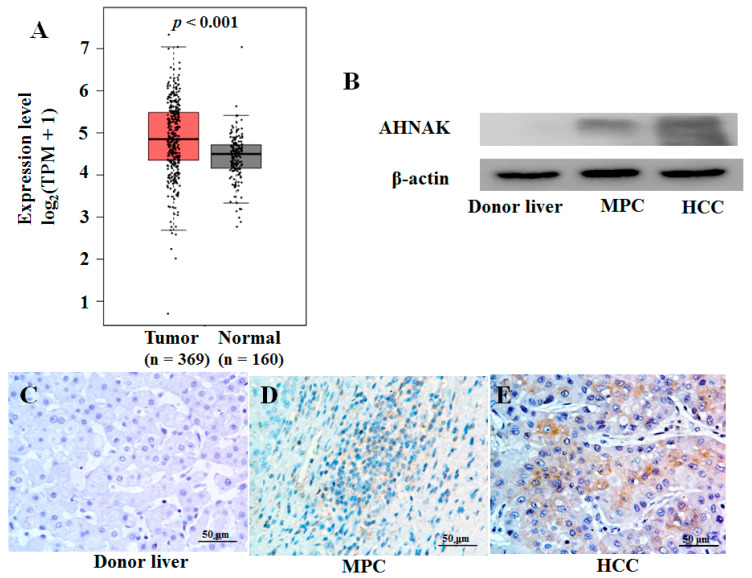
AHNAK was overexpressed in HCC tissues: (**A**) Up-regulated mRNA expression of AHNAK in HCC tissues samples from TCGA database (369 tumor and 50 normal samples) and GTEx database (110 normal samples); *p* < 0.001. (**B**) Results of Western blotting analysis of protein expression of AHNAK in donor liver, tumor and MPC tissues. Results of immunohistochemical analysis of protein expression of AHNAK in donor liver (**C**), MPC tissues (**D**) and HCC tissues (**E**). Scale bar, 50 μm. TCGA, The Cancer Genome Atlas; GTEx, Genotype-Tissue Expression; MPC, matched paracancerous.

In previous studies [15], we found high-level mRNA expression of AHNAK in HCC tissue, which was consistent with the results of The Cancer Genome Atlas (TCGA) and the Genotype-Tissue Expression (GTEx) database (Figure 2A). Here, we analyzed the expression of AHNAK protein in HCC and MPC tissues. We found that the AHNAK protein expression level in tumor tissues was higher than that in MPC tissues, and the results were confirmed with Western blotting (Figure 2B) and immunohistochemistry (Figure 2C–E). These results showed that AHNAK overexpressed both in HCC and MPC tissues compared with donor liver tissues. The presence of field cancerization has been confirmed in HCC [16,17]. The cancer-promoting AHNAK expressed in HCC field cancerization might be a new opportunity worthy of study to explore the signature of HCC field cancerization and to comprehend the important role that AHNAK plays in HCC development and progression.

### 2.3. Identification of AHNAK as a Candidate Involved in IGF-1 Signaling Transduction

**Figure 3:**
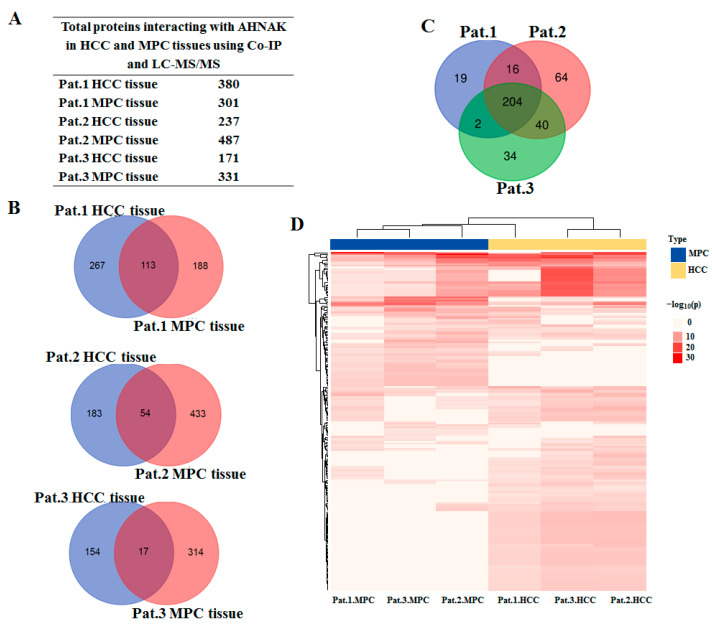
AHNAK involving pathways identified using LC-MS/MS: (**A**) Total proteins interacting with AHNAK in HCC and MPC tissues using Co-IP and LC-MS/MS. (**B**) Venn diagram graphic showing common and specific protein interaction with AHNAK between HCC and MPC tissues from Pat.1, Pat.2 and Pat.3. (**C**) Venn diagram graphics showing 204 enriched pathways shared by all three HCC patients (*p* < 0.001). (**D**) Heatmap showing the 204 enrichment pathways in HCC and MPC tissues. The discrete red scale represents statistical significance, and gray indicates a lack of significance. LC-MS/MS, liquid chromatography coupled with tandem mass spectrometry; Co-IP, co-immunoprecipitation; MPC, matched paracancerous.

The HCC field cancerization concept proposes that the paracancerous tissues of HCC patients are at a high risk of HCC [18]. To determine the role of AHNAK in the field cancerization of HCC, we analyzed the AHNAK interactome in HCC and MPC tissues. Three HCC tissues and their MPC tissues were tested using endogenous Co-IP and LC-MS/MS. To reduce false positives, Co-IP was performed on samples using antibody isotype IgG as control. Therefore, a total of 12 samples were tested using LC-MS/MS, and their total ion current chromatograms are shown in Supplementary Materials: Figure S1. By comparing the results of anti-IgG and anti-AHNAK, the proteins that could not be detected in the anti-IgG samples but could be detected in the anti-AHNAK ones were selected as the total interaction proteins of AHNAK in the samples. The total and specific non-redundant proteins interacting with AHNAK in HCC and MPC tissues of three HCC patents are shown in Figure 3A,B. The protein functional annotation and enrichment of signaling pathways were analyzed with the Reactome Gene Sets in Metascape platform. The enriched pathways using proteins interacting with AHNAK both in HCC and MPC tissues from Pat.1, Pat.2 and Pat.3 were 241, 324 and 280, respectively. The intersection of three Venn diagrams identified 204 enriched pathways shared by all three HCC patents (*p* < 0.001) (Figure 3C). The AHNAK protein involving 204 enriched pathways could be mainly divided into 10 categories: cellular signal transduction, cell cycle, cellular response to stress, degradation of proteins, metabolism of biological macromolecules, citric acid cycle, apoptosis, transport of proteins and small molecules, transcription and translation, and immune response. As can be seen in the heat map, the 204 enrichment pathways involving AHNAK protein were different comparing HCC with MPC tissues, which suggested that AHNAK might perform different functions in MPC and HCC tissues (Figure 3D).

**Figure 4:**
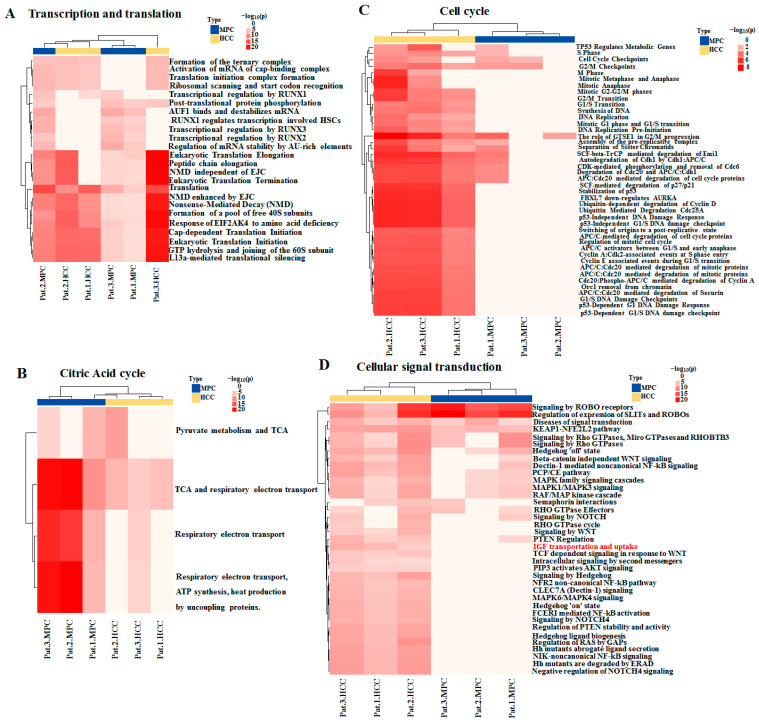
Heatmap showing enrichment pathways involving AHNAK protein in HCC and MPC tissues: (**A**) Transcription and translation. (**B**) Citric acid cycle. (**C**) Cell cycle; (**D**) Cellular signal transduction. Red text indicating enriched pathway: “IGF uptake and transportation”. MPC, matched paracancerous.

Of the 10 categories of enriched pathways and processes involving AHNAK protein, 4 are shown in Figure 4, and 6 are shown in supplementary Figure S2. The differences in the enriched statistical significance of apoptosis, cellular response to stress, metabolism of biomacromolecules, and transcription and translation (Figure 4A) were not obvious in MPC and HCC tissues, which suggests that HCC field cancerization might first occur in these pathways and processes. While most cell cycle pathways (Figure 4C), part of cellular signal transduction pathways (Figure 4D), the degradation of proteins, the transport of proteins and the immune response showed statistical significance in HCC tissues, it was not the same case in MPC tissues. In other words, these pathways were enriched in HCC tissues, while they were little or no enriched in MPC tissues. The results showed that these pathways involving AHNAK were activated in HCC but not in MPC tissues. In MPC tissues, citric acid cycle (Figure 4B) and respiratory chain processes were still in dominant positions among the energy generation sources. Taken together, these results indicated differences of AHNAK involved enrichment pathways and processes between HCC and MPC tissues and partially reflected the signature of HCC field cancerization.

It is known that giant AHNAK is located on the cell membrane and in the cytoplasm and it possesses an N-terminal PDZ-like region (postsynaptic density protein-95, disc large and zonula occludens-1) [19], which could interact with the C-terminal tail (serine-threonine-cysteine) of insulin-like growth factor 1 receptor (IGF-1R) [20]. IGF-1 is a ligand of IGF-1R, which is phosphorylated and activated when binding to the factor. According to the results of Co-IP, LC-MS/MS and previous studies [19,20], we hypothesize that AHNAK might bind to IGF-1R and subsequently has an effect on the transportation and uptake of IGF-1 or the activation of the receptor.

### 2.4. Identification of AHNAK as a Novel IGF-1R-Interacting Protein

**Figure 5:**
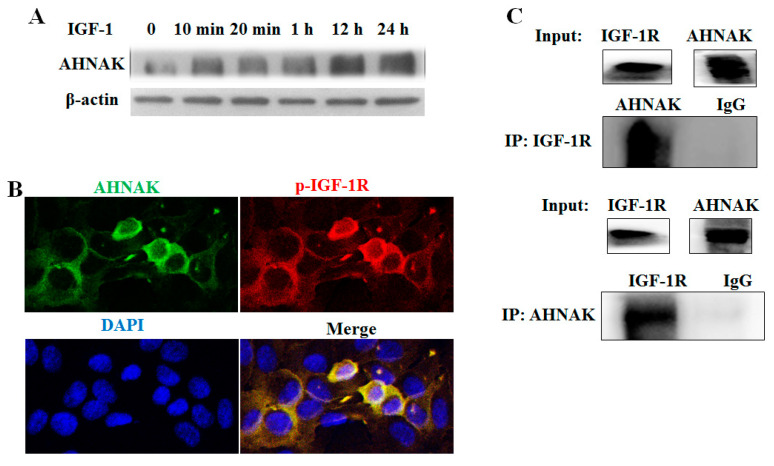
Identification of AHNAK as a novel IGF-1R-interacting protein: (**A**) Western blotting experiments showed that IGF-1 induced AHNAK expression. (**B**) Confocal microscopy scans of immunofluorescence double staining showed that AHNAK (green) co-localized with IGF-1R (red) in serum-starvation-treated HepG2 cells. (**C**) Co-immunoprecipitation and Western blotting experiments showed interactions between AHNAK and IGF-1R.

To test the hypothesis that AHNAK protein might interact with IGF-1R, serum-starved HepG2 cells were examined. HepG2 cells were starved overnight and were then stimulated with IGF-1 (50 ng/mL) for a time interval, as shown in Figure 5A, prior to harvesting. The Western blotting analysis of whole-cell extracts demonstrated that treatment with IGF-1 led to the potentiating of AHNAK expression. To further investigate the role of AHNAK in IGF-1 signaling transduction, immunofluorescence double staining and Co-IP were used to assess the interactions between AHNAK and IGF-1R. As shown in Figure 5B, HepG2 cells were starved overnight and were then stimulated with IGF-1 (50 ng/mL) for 1 h. Confocal microscopy analyses revealed that IGF-1 could induce AHNAK (green puncta) expression on the cell membrane and in the cytoplasm of serum-starved HepG2 cells; meanwhile, the result demonstrated the co-localization of AHNAK with phosphorylated IGF-1R (red puncta). Based on protein sequences, an online tool, PPA-Pred2, was used to make a prediction on the protein binding affinity between AHNAK and IGF-1R. The predicted value of Delta G (binding free energy) indicates spontaneous binding between two proteins, and the higher the absolute value of Delta G is, the more stable the binding affinity is. The value of Delta G was predicted to be −37.66 kcal/mol between AHNAK and IGF-1R using the “Miscellaneous” category. Then, the interaction was confirmed with Co-IP experiments. HepG2 cells were starved overnight and then stimulated with IGF-1 (50 ng/mL) for 1 h. Immunoprecipitation followed by Western blotting was performed to detect the interactions between AHNAK and IGF-1R. Our data showed that AHNAK was pulled down by anti-IGF-1R; meanwhile, IGF-1R was also pulled down by anti-AHNAK. Then, they were detected using the corresponding antibody (Figure 5C). These data verified the direct physical binding of AHNAK and IGF-1R in vitro, which suggested that the AHNAK complex promoted HCC growth, potentially by interacting with IGF-1R.

## 3. Discussion

AHNAK has been previously identified as a structural scaffold protein [5], implicated in a range of cancer-related pathways and processed as an ambiguous factor [7,8,11,14,17], which might depend on the type of tumor cells [21]. AHNAK has been described as a nucleoprotein that is significantly suppressed in neuroblastoma cell lines [22]. Immunoreaction to AHNAK has mainly been observed in the cytoplasm of normal cells compared with melanoma [10] and bladder urothelial carcinoma cells [22], whereas high levels of AHNAK have been shown to predict a poor outcome in PDAC [11], HCC [17] and laryngeal carcinoma [12]. AHNAK has also been reported to enhance metastasis in lung cancer [13] and mesothelioma [14]. Based on these observations, AHNAK might be a multifunctional protein and play variable roles in different types of cancers.

To investigate what interacted with AHNAK and its role in HCC, we performed Co-IP and LC-MS/MS using anti-AHNAK in HCC and MPC tissues, due to AHNAK being also expressed in MPC tissues. It has been shown that MPC tissues are not completely normal tissues, which is known as field cancerization and was first studied by Slaughter and Southwick [23]. It has been proposed that tumors originate from a field of mutation cells, such as aberrations of genetic, epigenetic or biochemical nature, which creates a permissive environment for malignant evolution, which could occur with or without morphological changes [24]. The molecular and cellular mechanisms underpinning the etiology of field cancerization remain largely unknown. In this study, AHNAK immunoreaction was observed both in MPC and HCC tissues, but not in donor liver. That proved to be an opportunity to explore the relationship between HCC field cancerization and the AHNAK interactome. The results showed that a total of 204 enriched pathways and processes involved by AHNAK in each HCC patient. Some pathways and processes, including transcription and translation, apoptosis, transport of proteins and small molecules, cellular response to stress and metabolism of biomacromolecules represented similar enrichment statistical significance both in HCC and MPC tissues, which suggests that AHNAK is involved in them both in MPC and HCC tissues. HCC field cancerization might first occur in these pathways and processes. The citric acid cycle and respiratory chain processes were still in dominant positions among the energy generation sources in MPC tissues. Generally, most types of cancer cells produce energy predominantly through the Warburg effect, which is a less efficient process of “aerobic glycolysis” consisting of a high level of glucose uptake followed by lactic acid fermentation taking place in the cytosol, not the mitochondria, and the preferential production of lactate even in the presence of abundant oxygen [25].

About half of the 204 pathways and processes mainly focusing on cell cycle, cellular signal transduction, degradation of proteins and immune response represented enrichment statistical significance in HCC tissues but a lack of significance in MPC tissues. AHNAK might perform more functions in HCC than in MPC tissues. In tumor cells, AHNAK mRNA was found to be significantly enriched in the G0 and G1 phases and substantially reduced in S/G2 of the cell cycle [26]. AHNAK serves as a G1-enriched interactor of p53-binding protein 1 (53BP1) to regulate the tumor cell cycle [26]. The AHNAK-53BP1 complex has been shown to suppress p53 target gene networks in multiple cancer types; meanwhile, AHNAK also directly interacts with p53 and inhibits p53-mediated target gene expression [27]. The concealed role of AHNAK in curbing the spontaneous activation of p53 response to fine-tune the levels of “G1-S checkpoint” has been identified. These results together indicate that high-level expression of AHNAK is relevant to cancer cell proliferation, in particular, that caused by a p53 function defect [26]. These results are consistent with our observation of AHNAK-enriched G0/G1- or p53-related pathways and processes in HCC tissues (Figure 4C).

IGF/IGF-1R signaling plays a crucial role in tumorigenesis, proliferation and metastasis through the regulation of multiple downstream signaling pathways, including PI3K/AKT and MAPK/ERK pathways [28]. In a previous study, following the analysis results from TCGA database, AHNAK and IGF-1 were selected as independent risk factors associated with the prognosis of patients with bladder urothelial carcinoma [29]. The activation of IGF-1R signaling was associated with poor prognosis in HBV-related HCC [30]. In this study, IGF-1 could intrude the overexpression of AHNAK in starvation-treated HepG2 cells. Additionally, the N-terminal PDZ-like region possessed by AHNAK could interact with the C-terminal tail (serine-threonine-cysteine) of IGF-1R [19,20]. We applied immunofluorescence double staining and Co-IP to confirm the hypothesis of the interaction between AHNAK and IGF-1R. In the future, we need to study whether AHNAK plays a key role in IGF-1R phosphorylation and IGF/IGF-1R signaling pathway activation.

## 4. Materials and Methods

### 4.1. Cell Culture

Hepatoma cell line HepG2 cells were maintained in Dulbecco’s Minimal Essential Medium (DMEM) supplemented with 10% fetal bovine serum, 50 units/milliliter of penicillin and 50 microgram/milliliter streptomycin sulfate. Then, they were incubated at 37 °C in a humidified atmosphere of 5% CO_2_. Prior to the addition of IGF-1 (Cell Signaling Technology, Danvers, MA, USA), cells were maintained overnight in serum-free medium.

### 4.2. Tissue Samples

In total, four HCC patients who were admitted at Beijing You’An Hospital, Capital Medical University, from 1 June 2013 to 30 December 2016 were recruited. Liver donor tissues, tumor tissues and MPC tissues located at least 2 cm from the tumor were obtained at the time of segmental surgical resection or liver transplantation. The diagnosis of HCC was confirmed with a histopathological examination, and medical records were reviewed for clinicopathological characteristics, according to the following 2012 EASL clinical practice guidelines: Management of chronic hepatitis B virus infection and Barcelona clinic liver cancer staging system. Exclusion criteria were as follows: less than 18 years old; co-infection with human immunodeficiency virus, hepatitis C virus or hepatitis D virus; coexistence of liver injury caused by drug intake, alcohol consumption or autoimmune hepatitis; pregnancy; lactation. This study was approved by the medical research ethics committee of Beijing You’An Hospital, Capital Medical University, and adhered to the 1975 Declaration of Helsinki.

### 4.3. CoIP for Nano-LC-MS/MS Analysis

Tissue lysate was centrifuged at 14,000× *g* for 15 min at 4 °C, and protein A agarose beads (50%) were added to the supernatant; then, it was shaken at 4 °C for 10 min on horizontal ice to remove non-specific foreign proteins and reduce the background. From the samples, we removed protein A beads after centrifuging at 14,000× *g* for 15 min at 4 °C. We slowly shook the mixture of antibody and tissue lysate at 4 °C overnight. We added 100 μL of protein A agarose beads to capture the antibody and its bound proteins, and we slowly shook the antigen–antibody mixture at 4 °C overnight. We then centrifuged the mixture at 14,000× *g* for 5 s, collected the agarose bead antibody complex, removed the supernatant, and washed with cooled phosphate-buffered saline (PBS) buffer for three times.

### 4.4. Trypsin Digestion

The samples were treated with 5 mM dithiothreitol (dissolved in 25 mM ammonium bicarbonate) at room temperature for 40 min for disulfide bond reduction. The samples were treated with 15 mM iodoacetamide (dissolved in 25 mM ammonium bicarbonate) and kept away from light at room temperature for 40 min. Trypsin was added in a ratio of 1:50 for protease digestion at 37 °C overnight. After the desalination process, the peptide sample eluted with the highly hydrophobic eluent was vacuumized for standby.

### 4.5. Liquid Chromatography-Tandem Mass Analysis

The extracted peptide sample was redissolved with 0.1% acetic acid and centrifuged at 13,000× *g* for 10 min for mass spectrometry analyses. Peptides were separated using liquid chromatography using an Acclaim Pep Map 100 column and an EASY-Spray column on an EASY-NLC 1000 system (ThermoFisher Scientific, Waltham, MA, USA). The loading sample volume was 3 μL. The gradient was generated using mobile phase A (0.1% formic acid in water) and mobile phase B (0.1% formic acid in acetonitrile). Mass spectrometry was performed using an Obitrap Fusion Lumos mass spectrometer (ThermoFisher Scientific). The spray voltages were set at 2.2 kV, and the heated capillary temperature was 270 °C. The parameters of the MS/MS scan were as follows: resolution, 60,000 at 400 *m*/*z*; maximum isolation time, 30 ms; normalized collision energy, 40%. Data-dependent MS/MS: up to top five most intense peptide ions from the preview scan in Obitrap.

### 4.6. Protein Identification and Annotation

The raw MS files were analyzed and searched against the Uniprot-Homo Sapiens protein sequence database using Maxquant [31]. The parameters were set as follows: the protein modifications were carbamidomethylation (C) (fixed) and oxidation (M) (variable); the enzyme specificity was set to trpsin; the maximum missed cleavages were set to 2; the precursor ion mass tolerance was set to 3 ppm; and MS/MS tolerance was 0.01 Da. Only highly confidently identified peptides were chosen.

The protein functional annotation and identification of signaling pathways of potential targets were performed using Gene Ontology (GO) and Kyoto Encyclopedia of Genes and Genomes (KEGG) enrichment analyses using the Metascape platform. The “ComplexHeatmap” software package was used in R (Version 3.6.3) for cluster analyses and visualization. The online prediction software Protein-Protein Affinity Predictor (PPA-Pred2) is available at https://www.iitm.ac.in/bioinfo/PPA_Pred/ (accessed on 19 May 2022).

### 4.7. Immunoprecipitation (IP) and Western Blotting Analysis

Cells were lysed, and proteins were extracted and quantified. The cell lysates were incubated with specific antibodies or the anti-IgG control for 3–4 h at 4 °C with rotation. Then, samples were incubated with protein A/G agarose beads (Santa Cruz Biotechnology, Santa Cruz, CA, USA) overnight at 4 °C with rotation. The beads were pelleted using centrifugation at 1000× *g* for 30 s at 4 °C; then, they were boiled for 10 min at 95 °C and resolved with 10% SDS-polyacrylamide gel electrophoresis. The separated proteins were transferred to PVDF membranes (Millipore, Bedford, MA, USA), and the membranes were blocked in 5% skim milk and incubated overnight at 4 °C with anti-AHNAK or anti-IGF-1R at a dilution of 1:200. After washing with TBST, the membranes were incubated with a horseradish peroxidase-conjugated secondary antibody for 1 h at room temperature. Finally, the membranes were incubated with enhanced chemiluminescent reagents (Millipore) and exposed to autoradiography film in the dark.

### 4.8. Immunohistochemistry Array

The slides were dewaxed, rehydrated, and blocked with 3% hydrogen peroxide, and antigen retrieval was performed with citrate buffer (Dako target retrieval solution; citrate buffer at pH 6.0). Slides were incubated with AHNAK antibody at a dilution of 1:50 (Santa Cruz Biotechnology) overnight at 4 °C. Then, the slides were incubated with secondary antibody and daminobenzidine after washing with phosphate-buffered saline.

### 4.9. SgRNA Plasmid Constructs and Generation of a Stable AHNAK-Knockout HepG2 Cell Line

AHNAK was knocked out using the CRISPR/cas9 method. In brief, the main steps were as follows: First, four sgRNAs were designed: sgCtrl (5′-CCTCGTTCACCGCCGTCGCG-3′), sgAHNAK-1 (5′-TCTTCGTGGTGTAGATGCGC-3′), sgAHNAK-2 (5′-CCATCTTCCGACTTCAGCCG-3′) and sgAHNAK-3 (5′-CTGAAAGGCCCTAACGTAAA-3′). The primers were inserted into the sgRNA backbone to construct the lentivirus plasmid vectors. Lentiviral production was performed by transfecting the lentivirus vectors into HEK 293H cells and collecting the resulting supernatant after 48 h. Titers were determined by detecting the WPRE sequence that was located on the lentiviral vector and could be integrated into the cell genome with a target gene with a qRT-PCR assay. HepG2 cells were infected with sgCtrl, and AHNAK sgRNA-1, -2 and -3 lentiviruses and were screened using basticidin resistance.

### 4.10. Cell Viability Assays

CCK8 assays were performed to determine cell proliferation. An equal number of the indicated cells in 200 μL of culture medium was seeded into 96-well plates for 24 h. At the indicated time, 20 μL of CCK-8 working solution (Boster, Wuhan, China) was added into each well and incubated with culture medium at 37 °C for 2 h. Cell viability was determined using an absorbance value of OD450 nm on a microplate reader.

### 4.11. Confocal Immunofluorescence

Briefly, cells on chamber slides were fixed with 4% paraformaldehyde and permeabilized with 0.2% TritonX-100. The samples were probed with specific antibodies against AHNAK (Santa Cruz) and p-IGF-1R (Cell Signaling Technology) at 4 °C for 12 h and then incubated with FITC- and TRITC-labeled secondary antibodies (1:200 dilution) for 1 h. After each step, cells were washed two times using PBS. The cell nuclei were stained with DAPI for 3 min, and a Leica DM14000B confocal microscope was used to capture images.

## 5. Conclusions

In conclusion, our results partially reflected the signature of HCC field cancerization and showed that AHNAK promoted HCC growth, potentially by interacting with IGF-1R. We performed Co-IP and LC-MS/MS on HCC and MPC tissues to explore the relationship between HCC field cancerization and the AHNAK interactome. The results of immunofluorescence double staining and Co-IP confirmed the interaction between AHNAK and IGF-1R. The role of AHNAK in IGF-1R phosphorylation and IGF/IGF-1R signaling pathway activation is an important research direction.

## Data Availability

Not applicable.

## Supplementary Materials

The following supporting information can be downloaded at: https://www.mdpi.com/article/10.3390/molecules27248680/s1, Figure S1: Total ion current chromatograms of 12 samples tested with LC-MS/MS, Figure S2: Six categories of enriched pathways and processes involving AHNAK protein.
