# Supplementary material for: AHNAK Contributes to Hepatocellular Carcinoma Growth by Interacting with IGF-1R
Source: Molecules. 2022 Dec 8;27(24):8680. doi: 10.3390/molecules27248680 (PMC9782793; doi:10.3390/molecules27248680)
Supplement: Supplementary file 1 [file molecules-27-08680-s001.zip › supplementary Figure 2 .pdf]

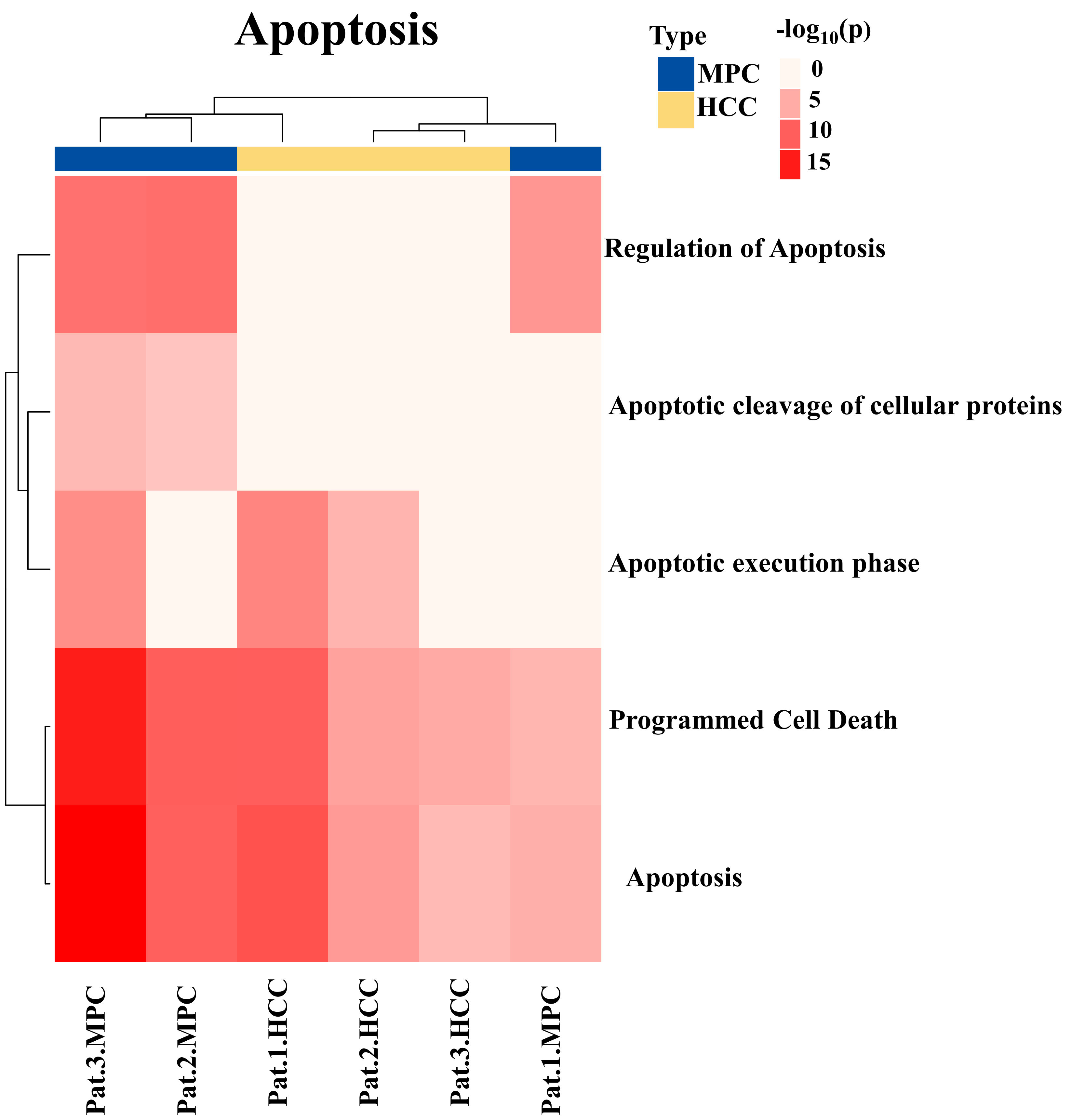
Apoptosis
Type
MPC
HCC
$-log_{10}(p)$
0
5
10
15
Regulation of Apoptosis
Apoptotic cleavage of cellular proteins
Apoptotic execution phase
Programmed Cell Death
Apoptosis
Pat.3.MPC
Pat.2.MPC
Pat.1.HCC
Pat.2.HCC
Pat.3.HCC
Pat.1.MPC

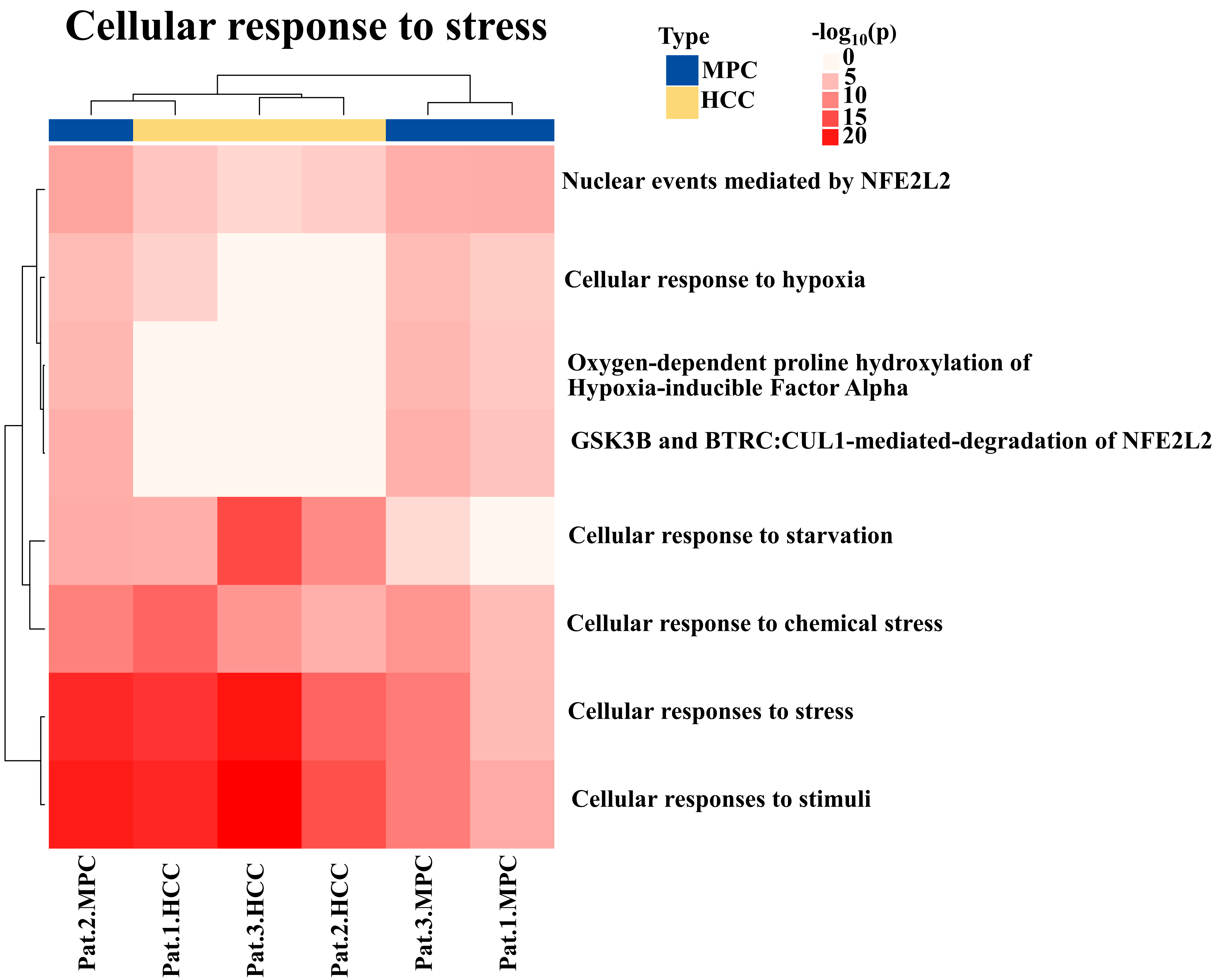

Cellular response to stress
Type
MPC
HCC
-log10(p)
0
5
10
15
20
Nuclear events mediated by NFE2L2
Cellular response to hypoxia
Oxygen-dependent proline hydroxylation of Hypoxia-inducible Factor Alpha
GSK3B and BTRC:CUL1-mediated-degradation of NFE2L2
Cellular response to starvation
Cellular response to chemical stress
Cellular responses to stress
Cellular responses to stimuli
Pat.2.MPC
Pat.1.HCC
Pat.3.HCC
Pat.2.HCC
Pat.3.MPC
Pat.1.MPC

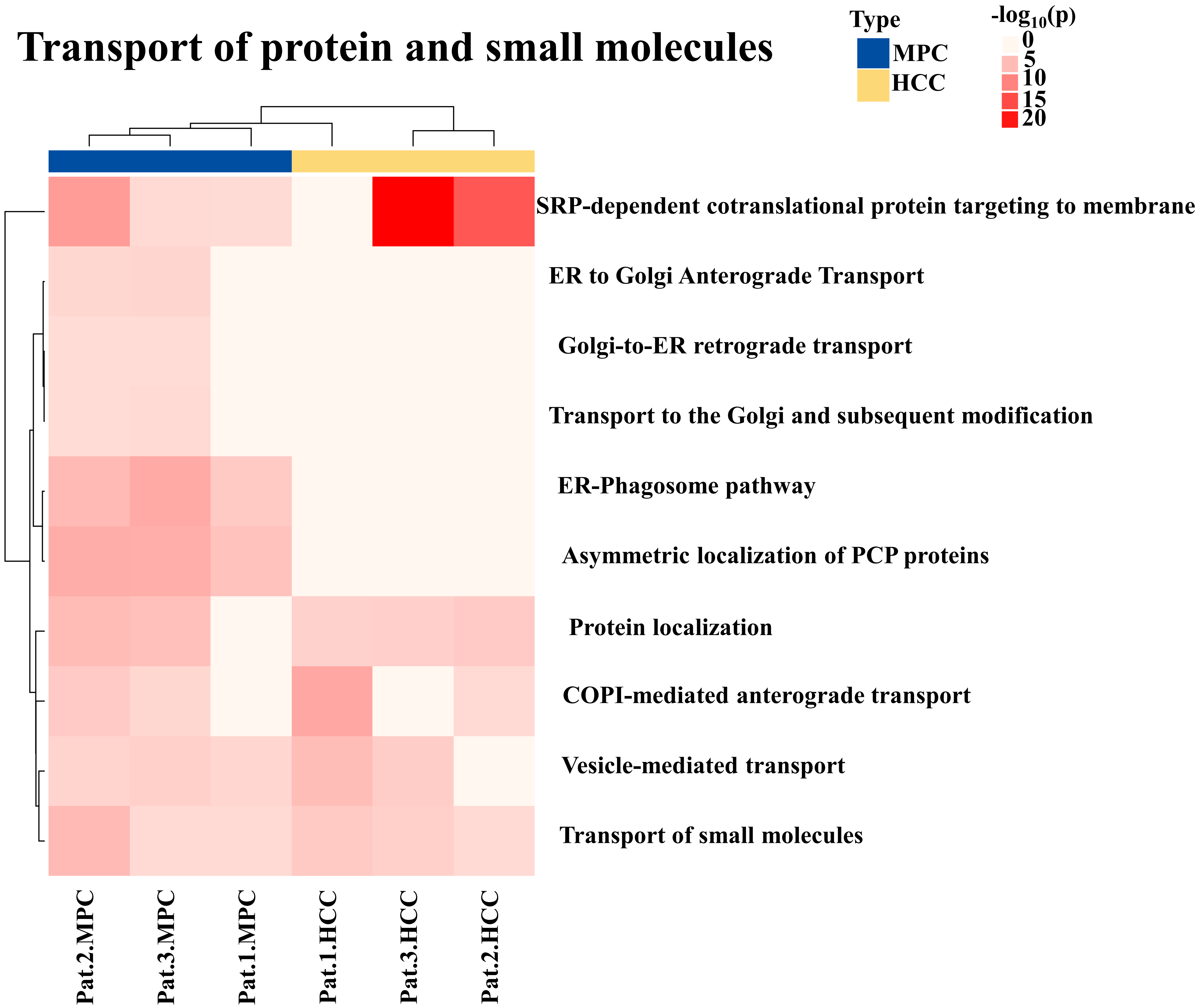
Transport of protein and small molecules
Type
MPC
HCC
$-\log_{10}(p)$
0
5
10
15
20
SRP-dependent cotranslational protein targeting to membrane
ER to Golgi Anterograde Transport
Golgi-to-ER retrograde transport
Transport to the Golgi and subsequent modification
ER-Phagosome pathway
Asymmetric localization of PCP proteins
Protein localization
COPI-mediated anterograde transport
Vesicle-mediated transport
Transport of small molecules
Pat.2.MPC
Pat.3.MPC
Pat.1.MPC
Pat.1.HCC
Pat.3.HCC
Pat.2.HCC

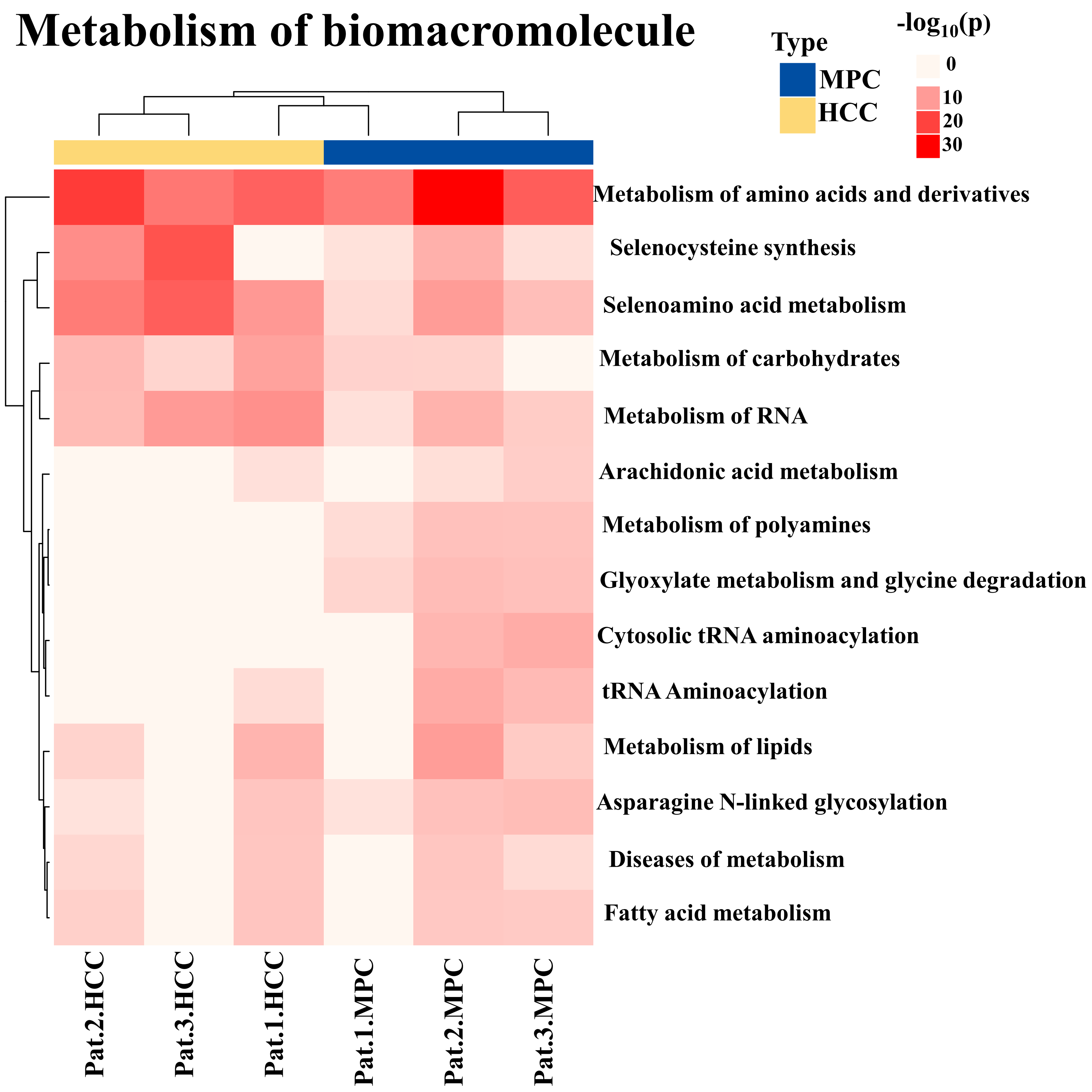
Metabolism of biomacromolecule
Type
MPC
HCC
$-log_{10}(p)$
0
10
20
30
Metabolism of amino acids and derivatives
Selenocysteine synthesis
Selenoamino acid metabolism
Metabolism of carbohydrates
Metabolism of RNA
Arachidonic acid metabolism
Metabolism of polyamines
Glyoxylate metabolism and glycine degradation
Cytosolic tRNA aminoacylation
tRNA Aminoacylation
Metabolism of lipids
Asparagine N-linked glycosylation
Diseases of metabolism
Fatty acid metabolism
Pat.2.HCC
Pat.3.HCC
Pat.1.HCC
Pat.1.MPC
Pat.2.MPC
Pat.3.MPC

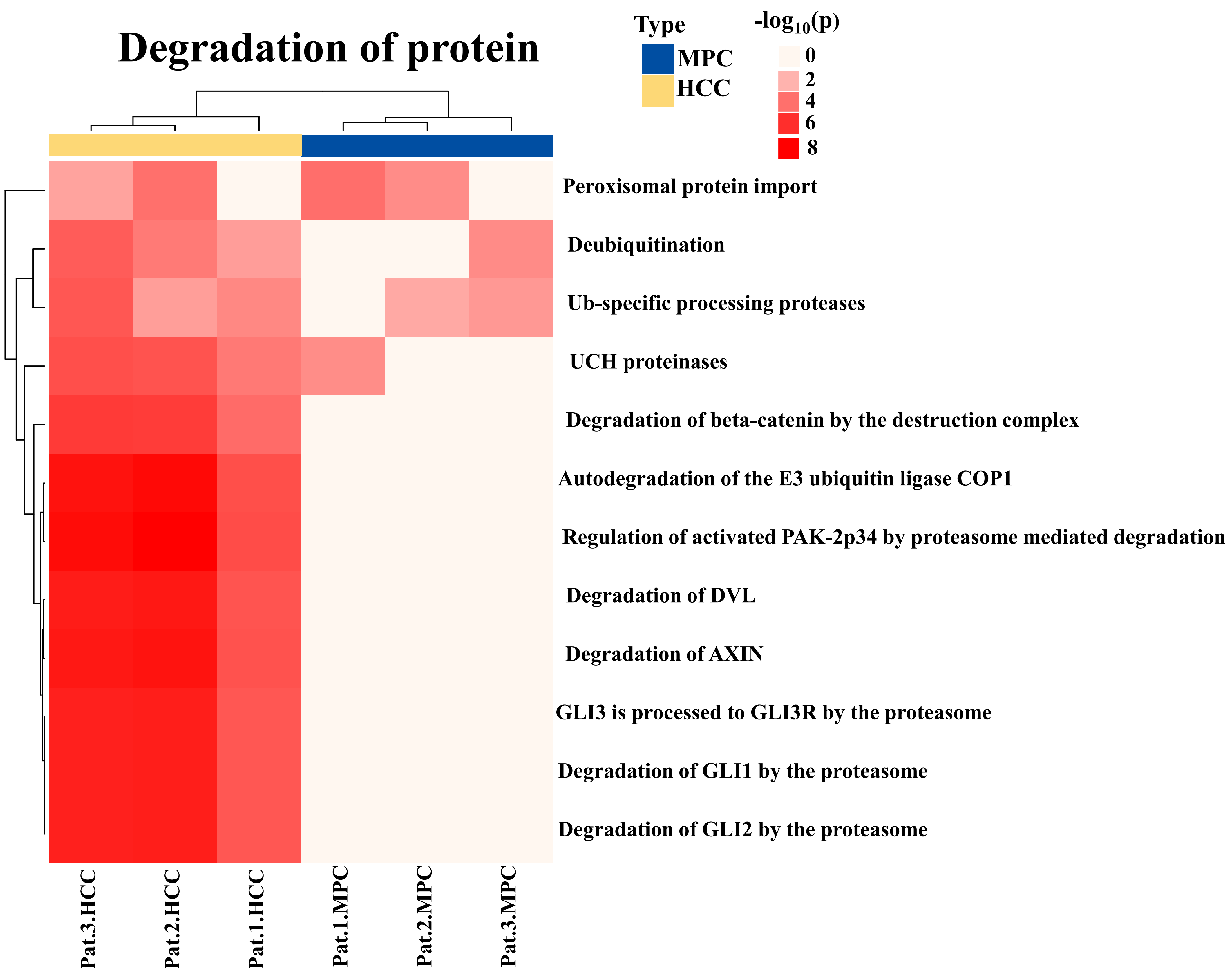

Degradation of protein
Type
MPC
HCC
-log10(p)
0
2
4
6
8
Peroxisomal protein import
Deubiquitination
Ub-specific processing proteases
UCH proteinases
Degradation of beta-catenin by the destruction complex
Autodegradation of the E3 ubiquitin ligase COP1
Regulation of activated PAK-2p34 by proteasome mediated degradation
Degradation of DVL
Degradation of AXIN
GLI3 is processed to GLI3R by the proteasome
Degradation of GLI1 by the proteasome
Degradation of GLI2 by the proteasome
Pat.3.HCC
Pat.2.HCC
Pat.1.HCC
Pat.1.MPC
Pat.2.MPC
Pat.3.MPC

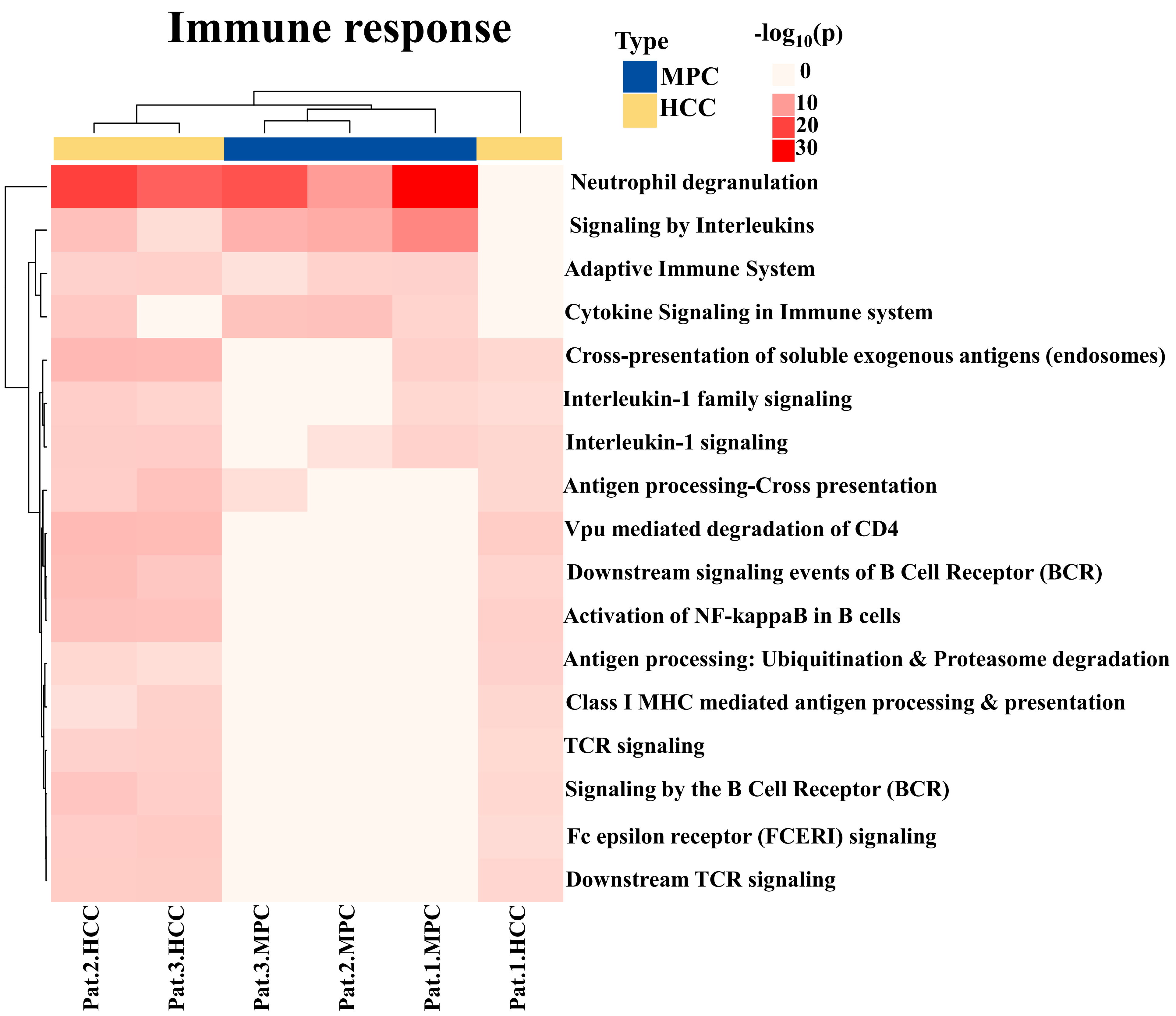
Immune response
Type
MPC
HCC
-log10(p)
0
10
20
30
Neutrophil degranulation
Signaling by Interleukins
Adaptive Immune System
Cytokine Signaling in Immune system
Cross-presentation of soluble exogenous antigens (endosomes)
Interleukin-1 family signaling
Interleukin-1 signaling
Antigen processing-Cross presentation
Vpu mediated degradation of CD4
Downstream signaling events of B Cell Receptor (BCR)
Activation of NF-kappaB in B cells
Antigen processing: Ubiquitination & Proteasome degradation
Class I MHC mediated antigen processing & presentation
TCR signaling
Signaling by the B Cell Receptor (BCR)
Fc epsilon receptor (FCERI) signaling
Downstream TCR signaling
Pat.2.HCC
Pat.3.HCC
Pat.3.MPC
Pat.2.MPC
Pat.1.MPC
Pat.1.HCC
