# Supplementary figures and images for: AHNAK Contributes to Hepatocellular Carcinoma Growth by Interacting with IGF-1R
Source: Molecules. 2022 Dec 8;27(24):8680. doi: 10.3390/molecules27248680 (PMC9782793; doi:10.3390/molecules27248680)

# Pat.1 HCC tissue, anti-AHNAK

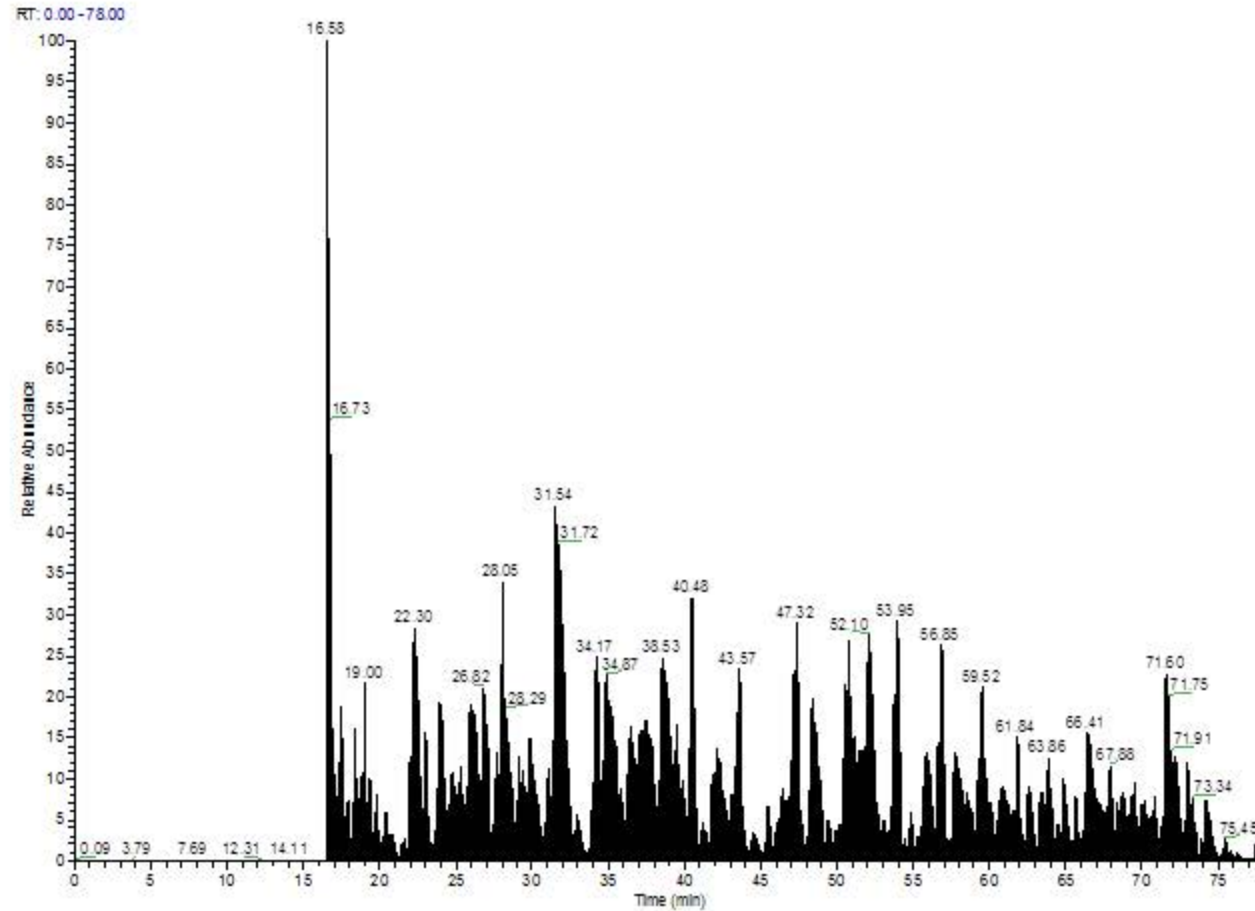

Supplement: Supplementary file 1 [file molecules-27-08680-s001.zip › supplementary Figure 1/1.pdf]

# Pat.3 HCC tissue, anti-IgG

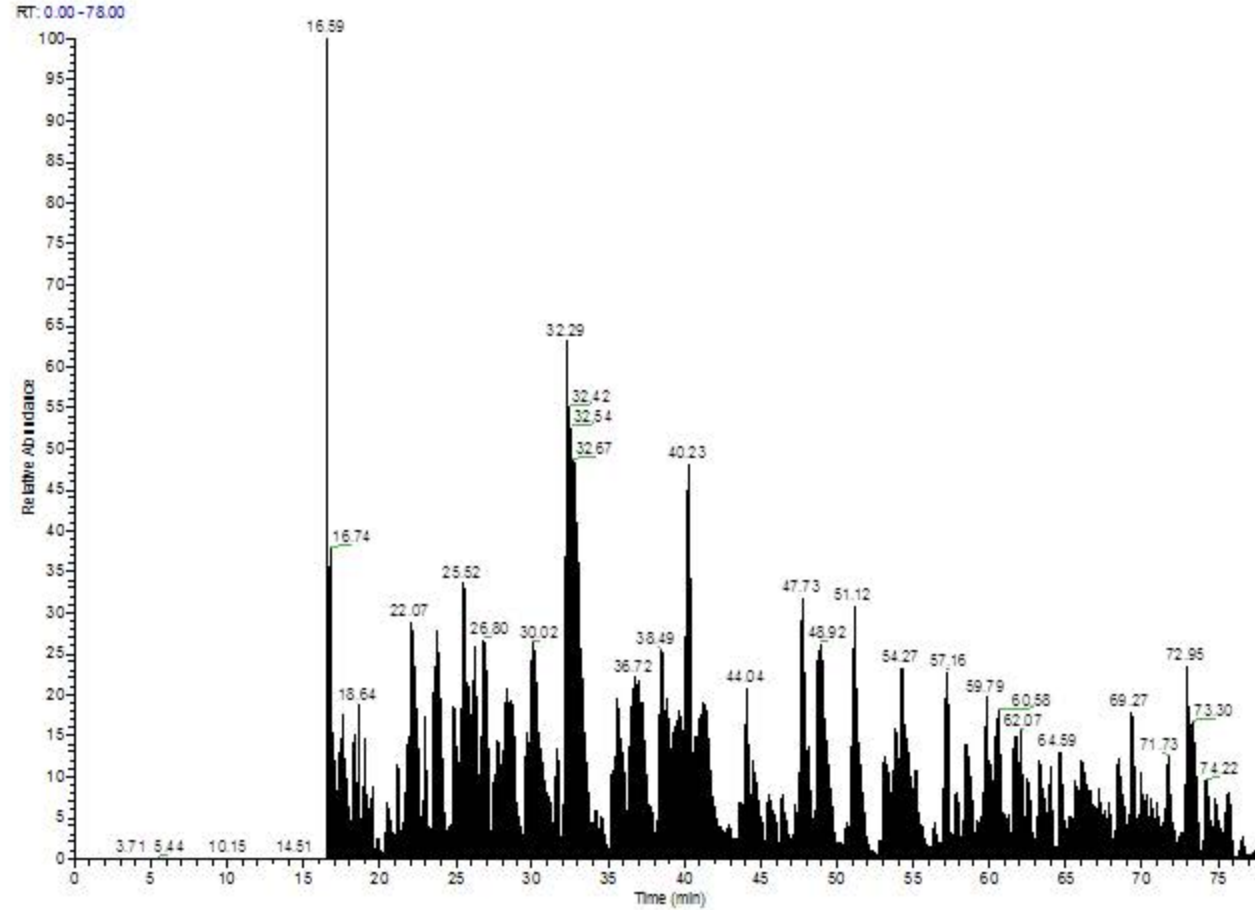

Supplement: Supplementary file 1 [file molecules-27-08680-s001.zip › supplementary Figure 1/10.pdf]

# Pat.3 paracancerous tissues, anti-AHNAK

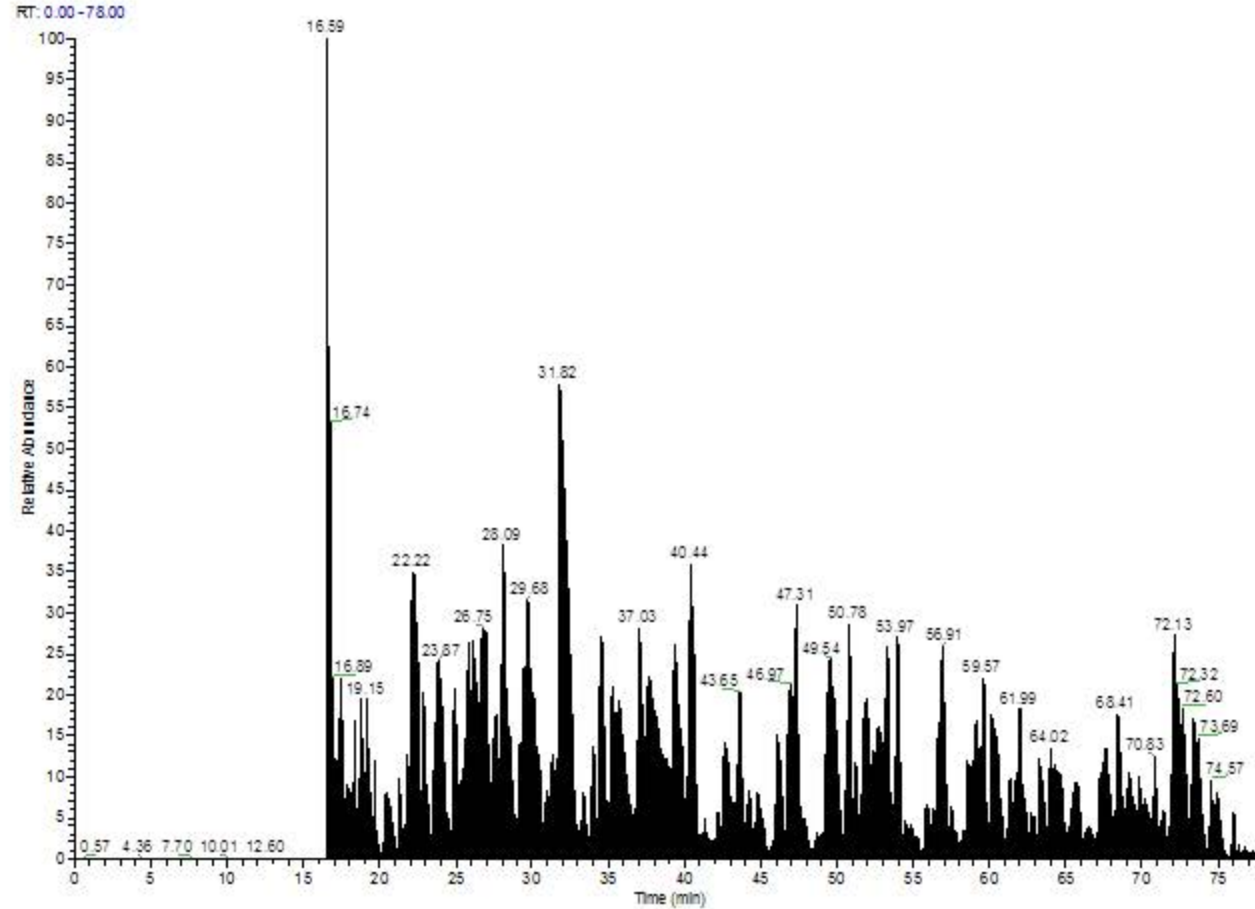

Supplement: Supplementary file 1 [file molecules-27-08680-s001.zip › supplementary Figure 1/11.pdf]

# Pat.3 paracancerous tissues, anti-IgG

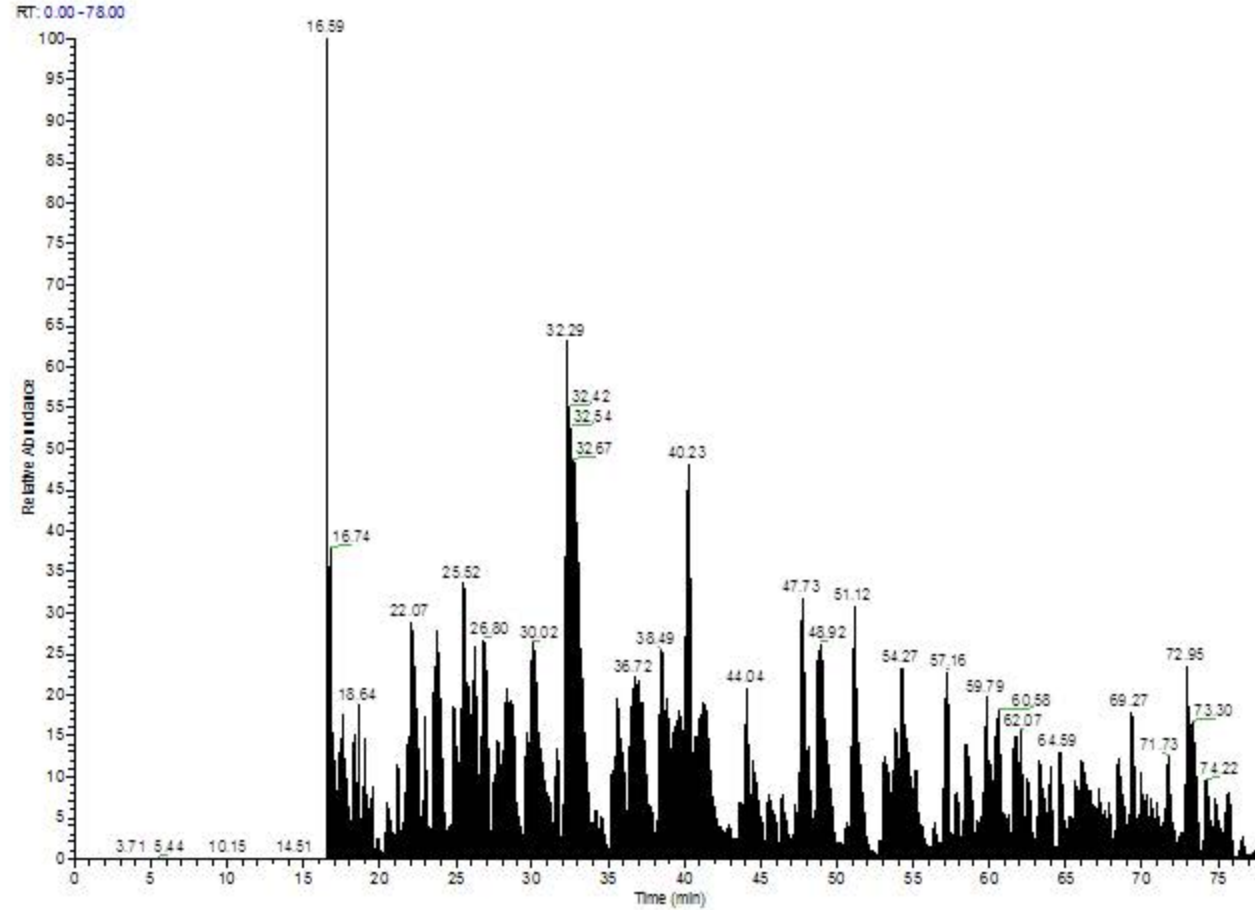

Supplement: Supplementary file 1 [file molecules-27-08680-s001.zip › supplementary Figure 1/12.pdf]

# Pat.1 HCC tissue, anti-IgG

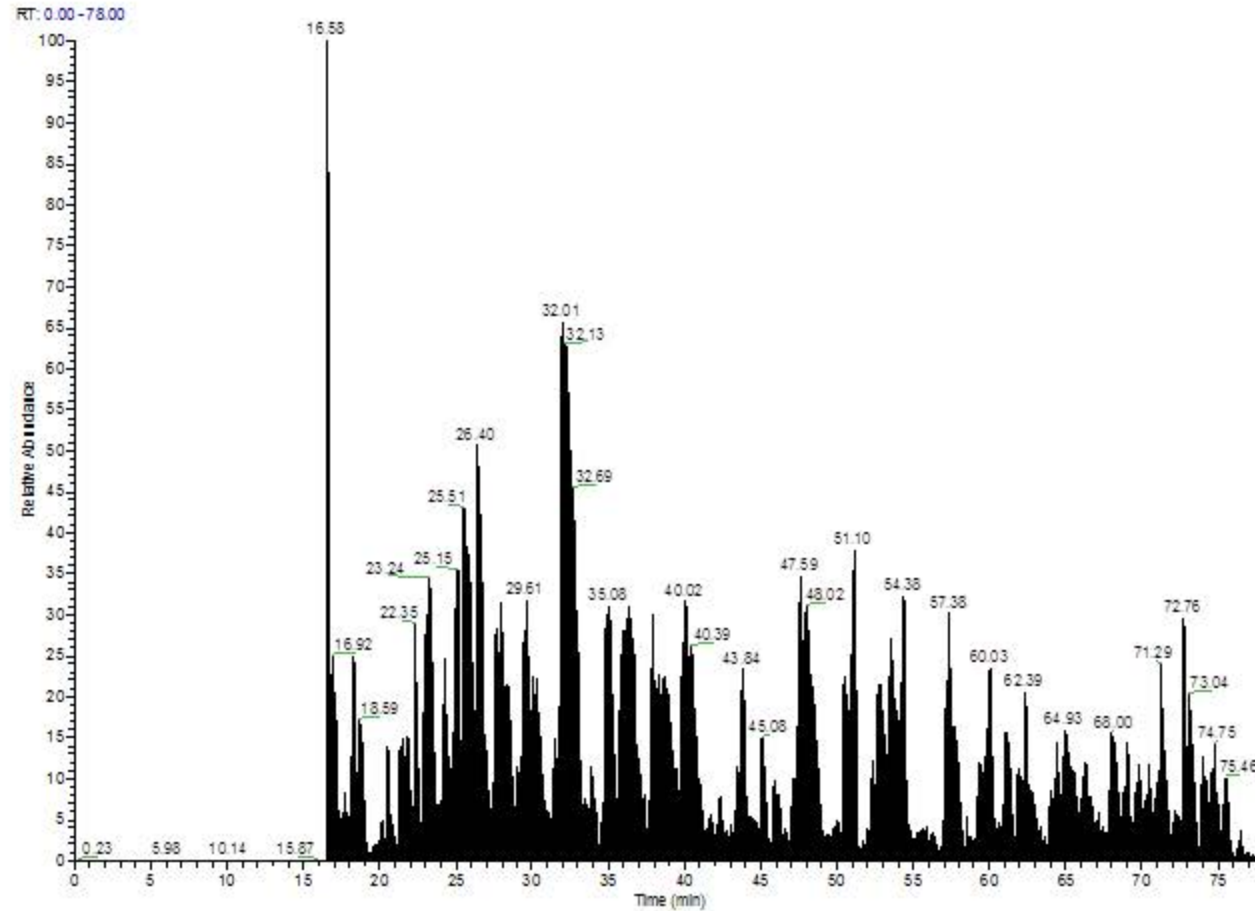

Supplement: Supplementary file 1 [file molecules-27-08680-s001.zip › supplementary Figure 1/2.pdf]

# Pat.1 paracancerous tissues, anti-AHNAK

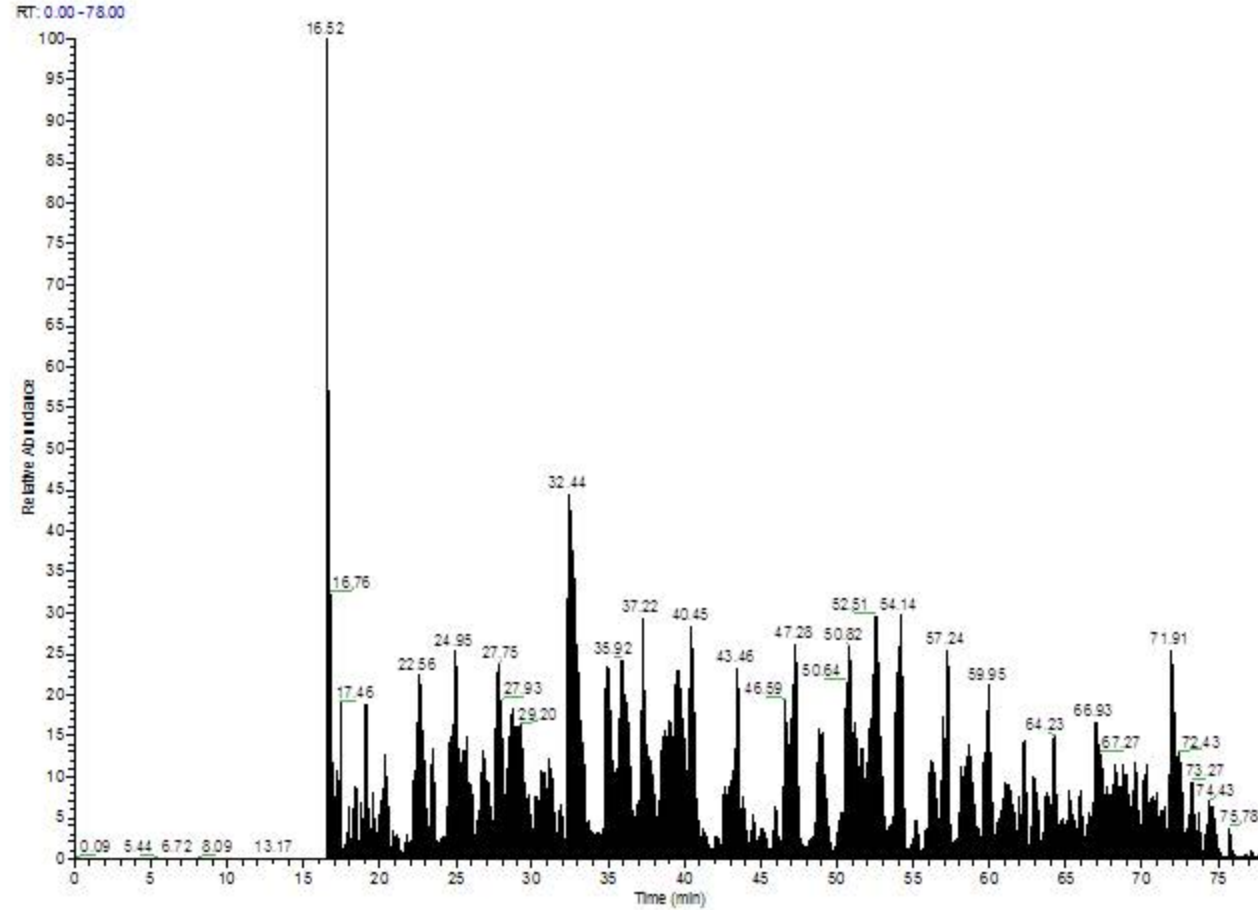

Supplement: Supplementary file 1 [file molecules-27-08680-s001.zip › supplementary Figure 1/3.pdf]

# Pat.1 paracancerous tissues, anti-IgG

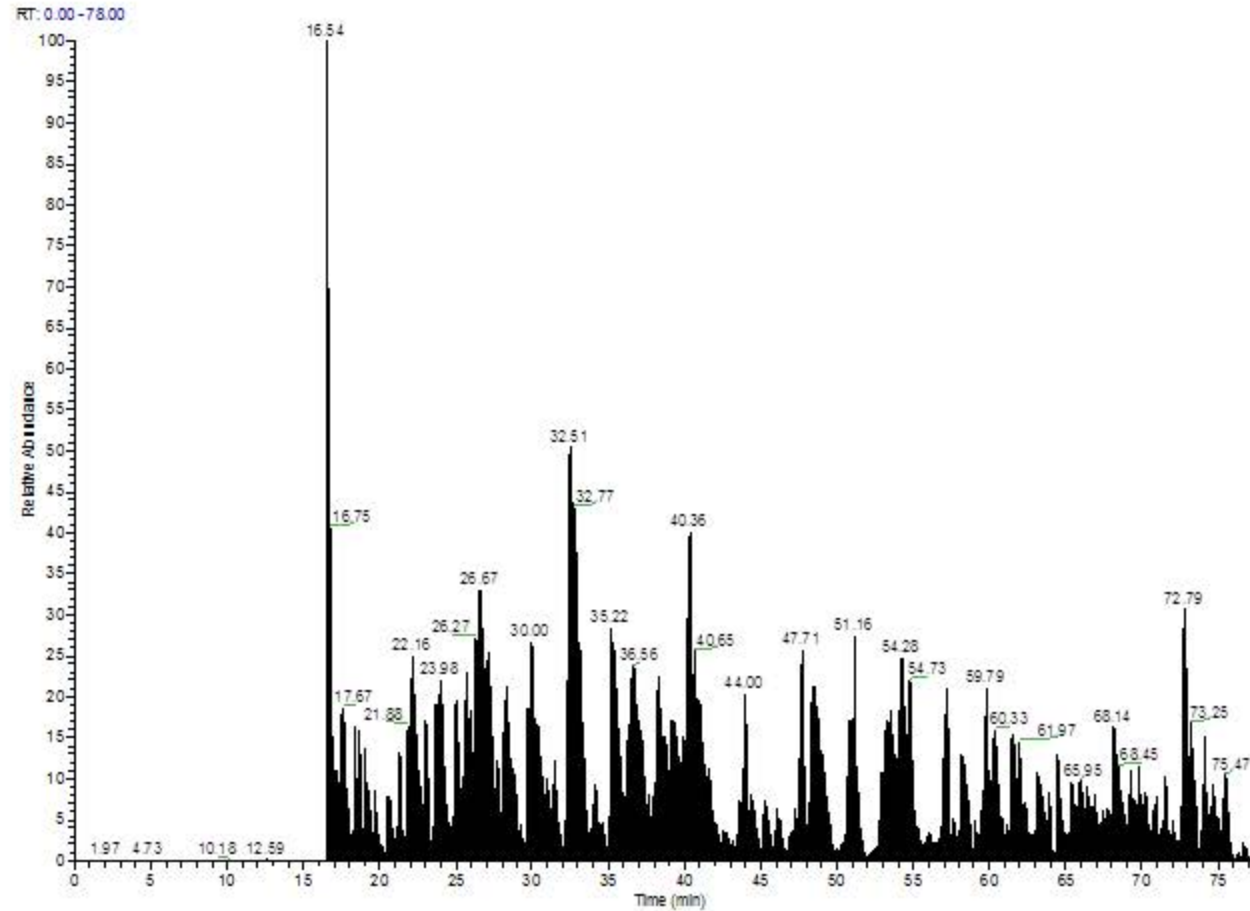

Supplement: Supplementary file 1 [file molecules-27-08680-s001.zip › supplementary Figure 1/4.pdf]

# Pat.2 HCC tissue, anti-AHNAK

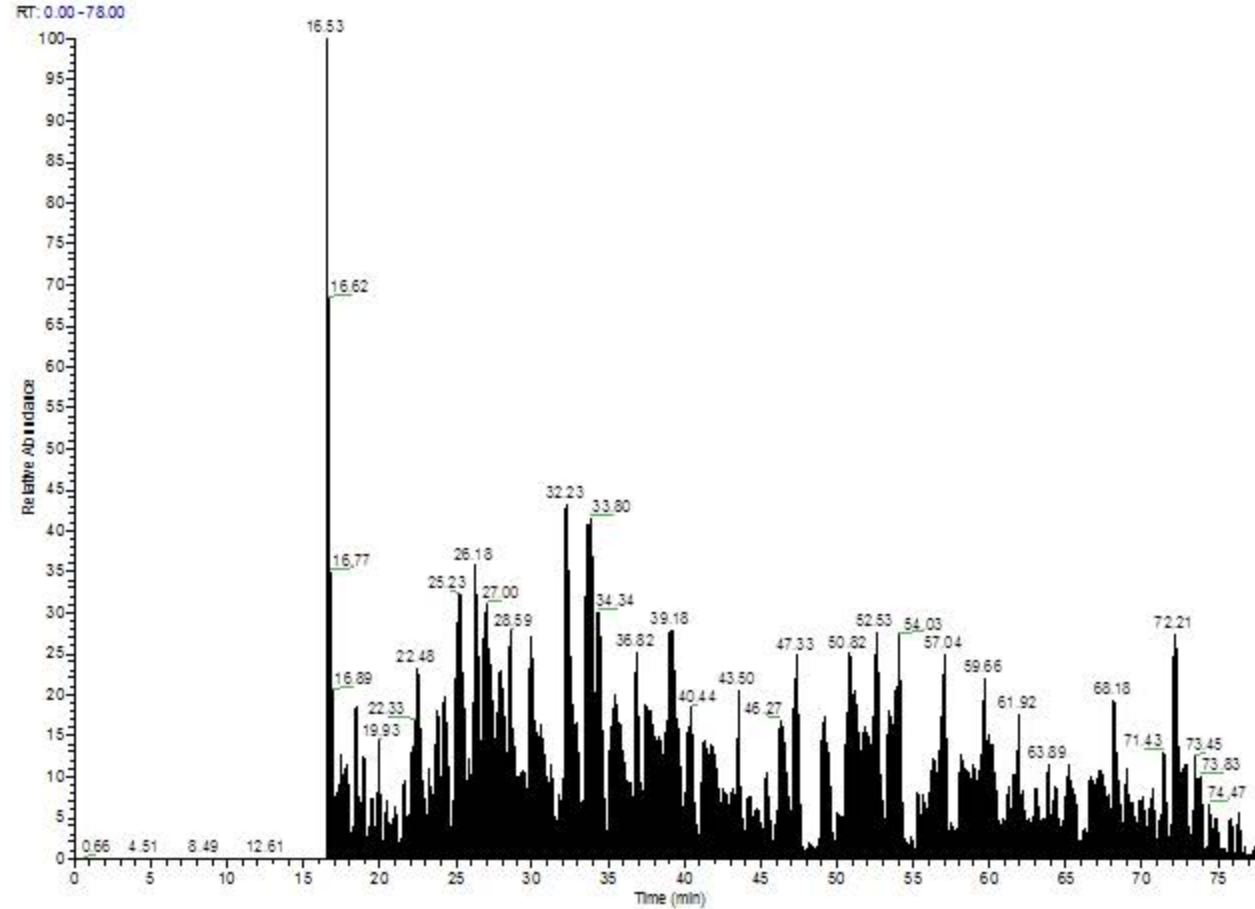

Supplement: Supplementary file 1 [file molecules-27-08680-s001.zip › supplementary Figure 1/5.pdf]

## Pat.2 HCC tissue, anti-IgG

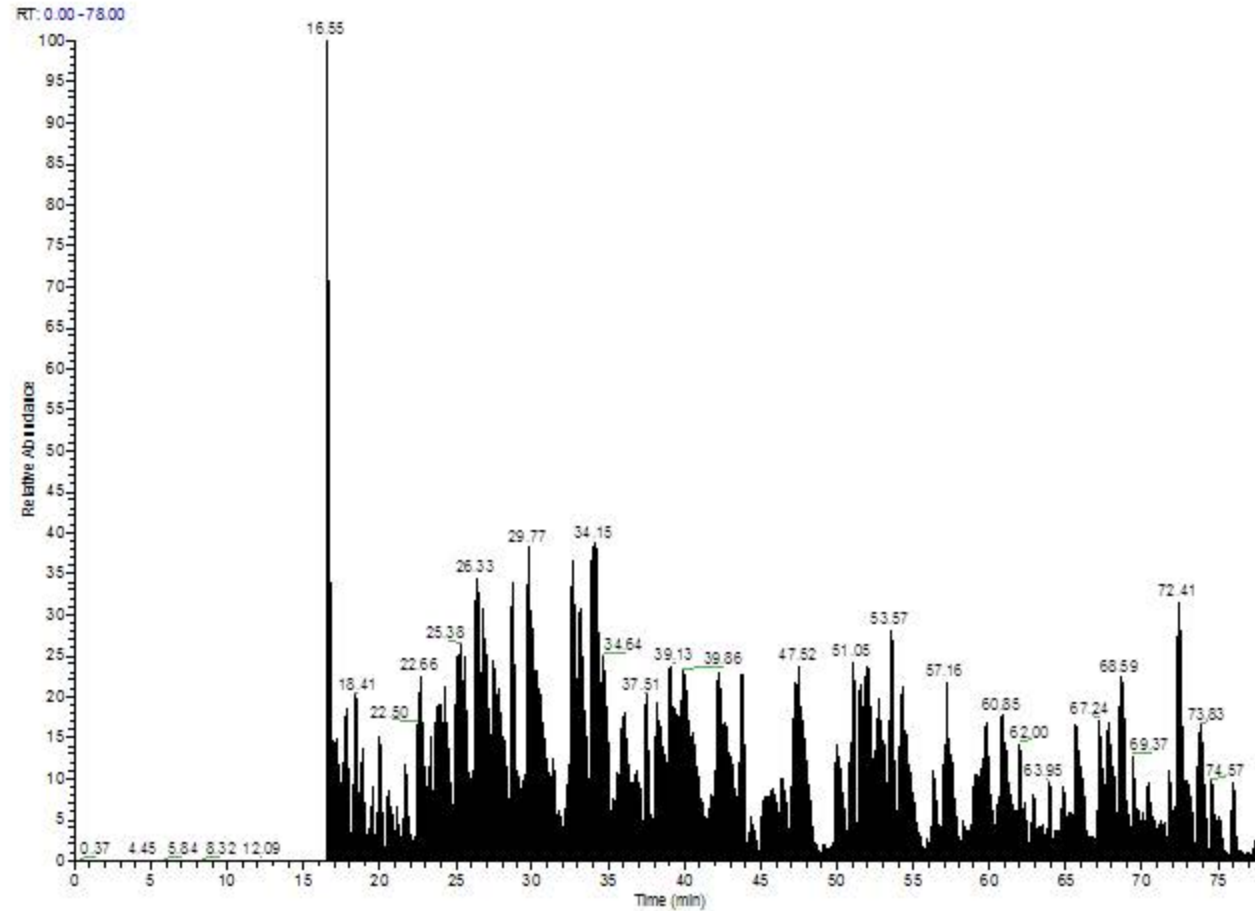

Supplement: Supplementary file 1 [file molecules-27-08680-s001.zip › supplementary Figure 1/6.pdf]

## Pat.2 paracancerous tissues, anti-AHNAK

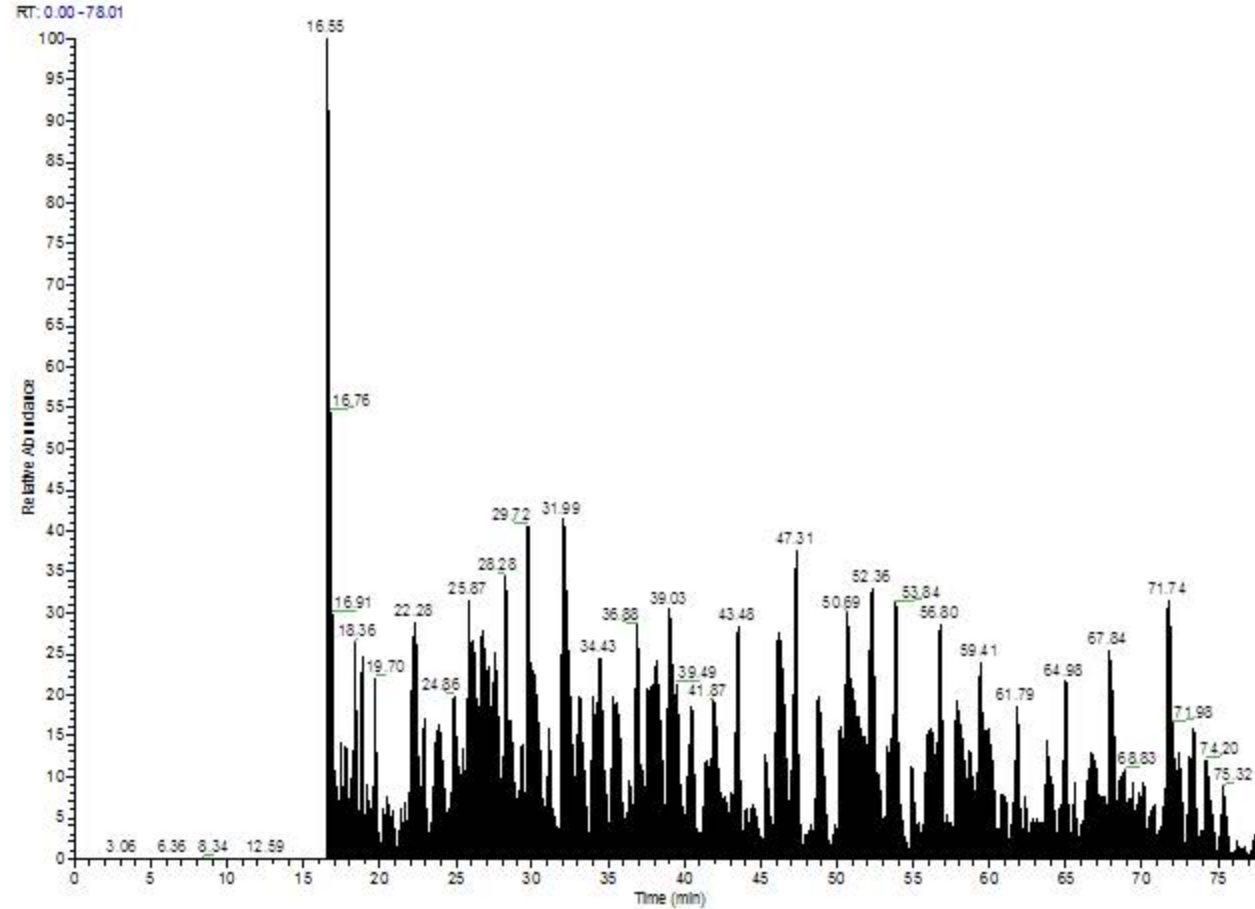

Supplement: Supplementary file 1 [file molecules-27-08680-s001.zip › supplementary Figure 1/7.pdf]

## Pat.2 paracancerous tissues, anti-IgG

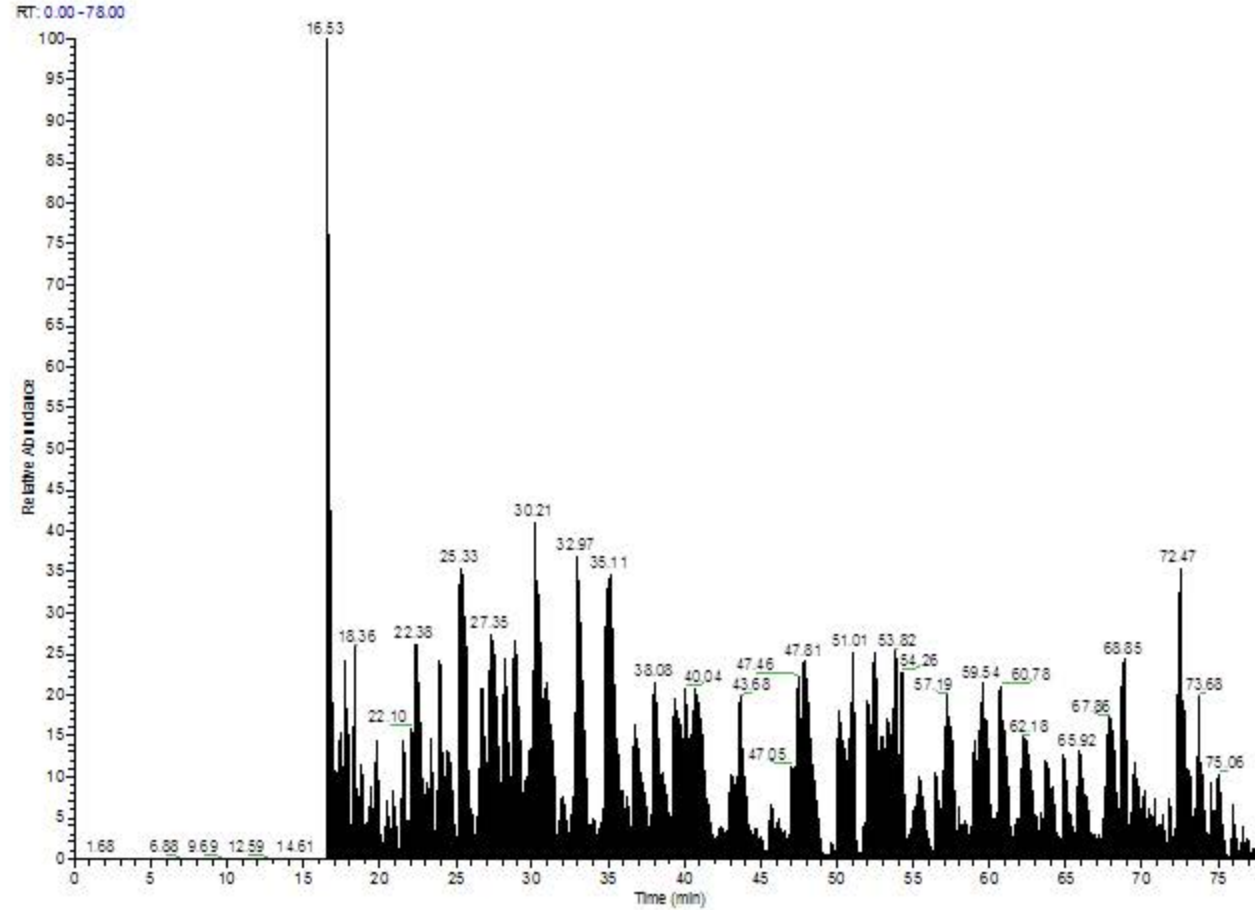

Supplement: Supplementary file 1 [file molecules-27-08680-s001.zip › supplementary Figure 1/8.pdf]

# Pat.3 HCC tissue, anti-AHNAK

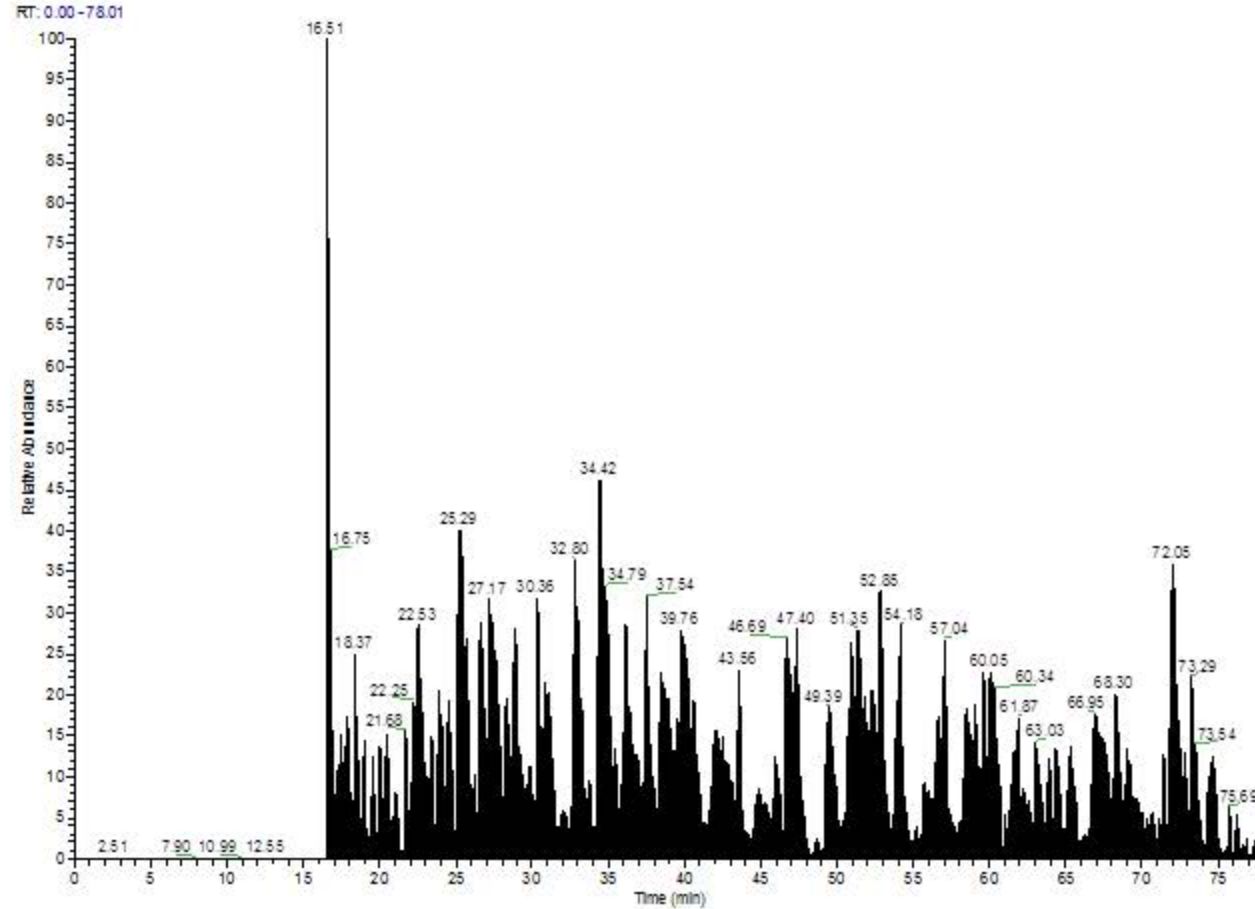

Supplement: Supplementary file 1 [file molecules-27-08680-s001.zip › supplementary Figure 1/9.pdf]
